# The Effects of Centralised and Specialised Intervention in the Early Course of Severe Unipolar Depressive Disorder: A Randomised Clinical Trial

**DOI:** 10.1371/journal.pone.0032950

**Published:** 2012-03-19

**Authors:** Hanne Vibe Hansen, Ellen Margrethe Christensen, Henrik Dam, Christian Gluud, Jørn Wetterslev, Lars Vedel Kessing, the Early Intervention Affective Disorders (EIA) Trial Group

**Affiliations:** 1 Mood Disorder Clinic, Psychiatric Centre Copenhagen, Rigshospitalet, Copenhagen University Hospital, Copenhagen, Denmark; 2 Copenhagen Trial Unit (CTU), Centre for Clinical Intervention Research, Rigshospitalet, Copenhagen University Hospital, Copenhagen, Denmark; Linkoping University, Sweden

## Abstract

**Background:**

Little is known on whether centralised and specialised combined pharmacological and psychological intervention in the early phase of severe unipolar depression improve prognosis. The aim of the present study was to assess the benefits and harms of centralised and specialised secondary care intervention in the early course of severe unipolar depression.

**Methods:**

A randomised multicentre trial with central randomisation and blinding in relation to the primary outcome comparing a centralised and specialised outpatient intervention program with standard decentralised psychiatric treatment. The interventions were offered at discharge from first, second, or third hospitalisation due to a single depressive episode or recurrent depressive disorder. The primary outcome was time to readmission to psychiatric hospital. The data on re-hospitalisation was obtained from the Danish Psychiatric Central Register. The secondary and tertiary outcomes were severity of depressive symptoms according to the Major Depression Inventory, adherence to medical treatment, and satisfaction with treatment according to the total score on the Verona Service Satisfaction Scale-Affective Disorder (VSSS-A). These outcomes were assessed using questionnaires one year after discharge from hospital.

**Results:**

A total of 268 patients with unipolar depression were included. There was no significant difference in the time to readmission (unadjusted hazard ratio 0.89, 95% confidence interval 0.60 to 1.32; log rank: χ^2^ = 0.3, d.f. = 1, p = 0.6); severity of depressive symptoms (mood disorder clinic: median 21.6, quartiles 9.7–31.2 versus standard treatment: median 20.2, quartiles 10.0–29.8; p = 0.7); or the prevalence of patients in antidepressant treatment (73.9% versus 80.0%, p = 0.2). Centralised and specialised secondary care intervention resulted in significantly higher satisfaction with treatment (131 (SD 31.8) versus 107 (SD 25.6); p<0.001).

**Conclusions:**

Centralised and specialised secondary care intervention in the early course of severe unipolar depression resulted in no significant effects on time to rehospitalisation, severity of symptoms, or use of antidepressants, but increased patient satisfaction.

**Trial Registration:**

ClinicalTrials.gov NCT00253071

## Introduction

Unipolar depressive disorder is associated with a high risk of relapse of depression and the risk of relapse increases as the number of previous episodes increases [1], [2]. Many patients do not recover to previous psychosocial function [3], some patients present with cognitive impairment also during remitted phase [4], and the risk of developing dementia seems to be increased in the long run [5], [6].

The tendency to relapse can be reduced by continued treatment with antidepressants [7], and potentially by cognitive behavioural therapy [8]. Nevertheless, results from naturalistic follow-up studies suggest that the progressive development of the diseases is not prevented in clinical practice with the present treatments [9]–[11]. Part of the explanation my be decreased adherence with antidepressants [12]–[15] and delayed intervention with pharmacological and psychological treatment programs. A number of studies have investigated the effect of combined or sequential pharmacological and psychological interventions [8] and various health-service interventions in recurrent depressive disorder [16]–[21] but none of studies have specifically investigated the effect in the early stage of severe depressive disorder. There are indirect suggestions that early intervention may improve the course and outcome. Antidepressants prescribed for depressive disorder may have neuroprotective abilities [22], [23] and patients may profit from psychotherapy before potential cognitive disturbances may occur during the long-term course of illness [24].

The aim of the present trial was to investigate the benefits and harms of centralised and specialised outpatient combined pharmacological and psychological intervention compared with standard psychiatric outpatient treatment early in the course of severe depressive disorder (unipolar disorder). The trial design was pragmatic with very few exclusion criteria and investigated the effect among patients following psychiatric hospitalisation in The Capital Region of Denmark. This pragmatic design was chosen to obtain a high generalisability of results from the trial to clinical settings regarding patients with the most severe depressive disorders [25].

## Methods

The protocol for this trial and supporting CONSORT checklist are available as supporting information; see Checklist S1 and Protocol S1.

### Trial design

The trial protocol has been described in details elsewhere [26]. A summary of the design and methods is presented in the following.

### Participants

A total of 268 patients with unipolar depression episode or recurrent depressive disorder were included from seven (Psychiatric Centre Hvidovre, Psychiatric Centre Copenhagen, Rigshospitalet; Psychiatric Centre Amager, Psychiatric Centre Frederiksberg, Psychiatric Centre Copenhagen, Bispebjerg, Psychiatric Centre Gentofte, Psychiatric Centre Hillerød) out of the nine psychiatric wards in The Capital Region of Denmark during a period from December 2005 to December 2009.

### Inclusion and exclusion criteria

#### Inclusion

Patients discharged from their first, second, or third hospitalisation from an inpatient psychiatric ward with an ICD-10 diagnosis of single moderate or severe depressive episode or recurrent depressive disorder (ICD-10 code: DF32.1–33.9) as the primary diagnosis. Comorbidity with alcohol or substance abuse and other psychiatric disorders were allowed. The physicians at the psychiatric wards diagnosed the patients. Age was between 18 and 70 year old. The patients were able and willing to give written and oral informed content.

#### Exclusion

Patients with moderate or severe dementia; with poor understanding of Danish; under any kind of commitment; or without informed consent were excluded.

### Randomisation

Patients were randomised to the intervention group or the control group at the end of the index hospitalisation while still in hospital. The Copenhagen Trial Unit conducted randomisation centrally according to a computer generated allocation sequence to secure allocation concealment. Allocation was stratified for psychiatric centre and number of hospitalisations for depression (1 or 2 compared to 3). The ratio of randomisation between the intervention and the control group was 1∶1. The randomisation was carried out with a block size of 20 unknown to the investigators.

### Blinding

Blinding of patients and the treating clinicians was not possible as patients were randomised to the mood disorder clinic or to standard treatment. The primary outcome was based on public register data using blinding for intervention. All other outcomes were assessed without blinding to the intervention. Two researchers (HVH and LVK) carried out all statistical analyses before the primary outcome data were unblinded.

### Experimental intervention group

Patients in the intervention group were treated in a specialised outpatient mood disorder clinic at the Psychiatric Centre Copenhagen, Copenhagen University Hospital, Rigshospitalet. The clinic was established September 2004 in parallel to the work and publication of a Health Technology Assessment (HTA) report on outpatient treatment in severe affective disorders [27].

The staff in the outpatient mood disorder clinic consists of full time specialists in psychiatry with specific clinical experience and knowledge on diagnosis and treatment of affective disorders as well as certified psychologists, nurses, and a social worker with experience in affective disorders.

During the first year following establishment of the clinic a detailed intervention program including manuals for psychological group interventions (psychoeducation and cognitive behavioural therapy) was developed, tested, and revised in a pilot phase with inclusion of approximately 30 patients. The manuals in Danish are available by contact to the last author (LVK). The final combined pharmacological and non-pharmacological intervention program was as follows. The intervention program lasted one year. According to the protocol, a medical doctor evaluated all patients in the clinic less than two weeks after discharge from the psychiatric inpatient ward. Although most patients improve during hospitalisation for depressive disorder, it is well known that they do still suffer from depressive symptoms at discharge from wards in the Copenhagen area [28]. Prior course of illness and effect of treatment was carefully recorded and diagnosis and treatment plans were re-evaluated and current pharmacological treatment adjusted in accordance with clinical status and national [29] and international guidelines [30], [31].

Subsequently, the physician at the mood clinic followed the patients with regular appointments depending on their clinical status and needs. In addition, patients participated in three different sequential group sessions weekly. All treatments sessions were carried out in accordance with manuals although individualised according to characteristics and needs of the patients in the group. The first group was a settling-in group for patients just discharged from hospitalisation. Here the focus was on the current clinical status and beliefs and experiences in relation to the recent hospitalisation. Patients stayed in this group until they were clinically stable and had at least partly remitted from depressive symptoms (HDS-17 items <14), i.e., typically for some months up to half a year. When stable, the patients were transferred to the second and intermediary group, consisting of either group psychoeducation or cognitive behavioural therapy. The type of group therapy was decided in concordance between the patient and the clinicians. These group sessions consist of 1½ hours intervention every week for 12 consecutive weeks. In both groups, focus was on knowledge and acceptance of suffering from a unipolar depressive disorder, identifying depressive symptoms from normal reactions, personal identity in relation to suffering from a depressive disorder, risk situations, stress management, the need for sustained pharmacological maintenance treatment, adverse effects to treatment, and identification of individual prior early warning signs of upcoming depressive episodes. In addition, the cognitive behavioural therapy group sessions focused on cognitive distortions in identity and behaviour and to some extend on inter-individual conflicts. Finally, the patients joined a 3–6 months discharge group that was a preparation for re-referral either to the general practitioner, a private psychiatrist, or to the community mental health centre with the aim of identifying individual early warning signals prospectively in practice and training of how to change upcoming personal conflicts and cognitive distortions.

Six to eight patients and two therapists (psychiatrist and psychologist or nurse) participated in each group. In the cognitive behavioural groups, at least one therapist had a formal education in cognitive behavioural therapy.

### Control group

The control group of patients were offered standard care consisting of the standard mental health service routines in The Capital Region of Denmark, i.e., treatment at the general practitioner, a private psychiatrist, or at the local community mental health centre. Participation in the trial had no influence on the treatment offered to these patients. Psychopharmacological treatment in the control group, compared with treatment in the mood disorder clinic, is likely more based on the preferences of the individual physician than on national and international guidelines. Psychosocial treatment elements like psychoeducation and cognitive behavioural therapy, and contact with family might be provided infrequently and in a less intensive, non-systematic way and presumably only for some patients as compared with the mood disorder clinic.

### Main outcome measures

The primary outcome was time to first readmission to psychiatric ward after discharge from the index hospitalisation (in the Trial registration report (www.ClinicalTrials.gov: ID: NCT00253071), the time frame to first readmission was specified to 2, 5, and 10 years, respectively, however, in the statistical analyses (see later) we included all follow-up time for all individuals in one primary analysis drawing more information from the available data).

Data on re-hospitalisation were obtained from the Danish Psychiatric Central Register that contains data on all inpatient and outpatient contacts to all psychiatric hospital-based services in Denmark [32]. Since 1 January 1994, the ICD-10 has been in use by the Register [33].

The secondary and the tertiary outcomes were assessed using questionnaires that were mailed to all participants one year after discharge from the index hospitalisation. The patients fulfilled the Major Depression Inventory (MDI) [34]–[36] and The Mood Disorder Questionnaire (MDQ) [37], [38] to measure severity of depressive symptoms and the prevalence of a hypomanic (or manic) episode. A hypomanic or manic episode was defined as a score of seven or more on the MDQ. When answering the MDI one year after discharge the patients were asked about depressive symptoms during the two weeks period with the worst symptoms within the last year. In addition, the patients were asked whether they were treated with maintenance medical treatment, antidepressant treatment, mood stabilizers, or antipsychotics.

The tertiary outcome was satisfaction with the intervention one year after discharge from the index hospitalisation estimated by the Verona Service Satisfaction Scale [39] adjusted to patients with affective disorder, Verona Service Satisfaction Scale-Affective Disorder (VSSS-A) [40] (inclusion of VSSS-A as a tertiary outcome was not specified in the trial registration report but in the trial protocol [26], which was published before inclusion of data and statistical analyses). VSSS-A includes among others 32 different items on satisfaction with care within seven different areas (overall satisfaction, professionals' skills and behaviour, information, access, efficacy, types of intervention, relative's involvement). Patients rate each item on a five-point Likert scale (1 = terrible, 2 = mostly dissatisfactory, 3 = mixed, 4 = mostly satisfactory, 5 = excellent) making it possible to calculate a mean total score of satisfaction (between 0–5).

### Sample size estimation

The sample size estimation has been described in detail elsewhere [26]. Based on results from the Health Technology Assessment (HTA) report on outpatient treatment in severe affective disorders [27], it was estimated that systematic outpatient combination treatment consisting of prophylactic pharmacotherapy and psychotherapy/psychoeducation compared to either medication alone or psychotherapy/psychoeducation alone could reduce the rate of recurrence of affective episodes between 14% and 44% and the rate of readmission with 35% during the first year after discharge from hospital. Anticipating a hazard ratio (HR) of 0.65 in the comparison of the intervention group with the control group on the primary outcome; a two-sided risk of type 1 error, α, of 0.05; a type 2 error risk, β, of 20%; (power of 80%) and equal group size, the sample size (N) for was calculated to N = 176 under the further assumption of a median time to re-hospitalisation of approximately 6 months in the control group, an inclusion period of 36 months, and a follow up period of 12 months. As the present trial ran in parallel to a trial aiming to include 176 patients with bipolar disorder, we decided to expand the inclusion period of the present trial with one year to a total of four years, as we had to expand the trial duration of the bipolar trial due to decreased recruitment.

The actual inclusion of 268 patients with unipolar depression over a four-year period results in a power of 93% for the detection or rejection of a HR of 0.65; of 82% to detect or reject a HR of 0.70; and of 63% to detect or reject a HR of 0.75.

### Statistical analysis

The statistical analyses were conducted as intention-to-treat analyses. Regarding the primary outcome, time to the first re-hospitalisation was estimated in a Kaplan-Meier plot, censoring at the date of death or end of study, and the difference in cumulated prevention of re-hospitalisation in the intervention and in the control group was tested in a log-rank test.

Hazard ratios (HR) adjusted for age, sex, psychiatric centre and number of previous psychiatric admissions were calculated in Cox' regression models.

Participants and non-participants were compared using register-based variables to evaluate whether participants in the trial were representative of patients with depressive disorder, discharged from their first, second, or third hospitalisation.

SPSS 19.0 for windows was used for the statistical analyses.

### Ethical considerations

The trial was approved by the Danish Research Ethical Committee (KF 01 272130), covering all hospitals in the region, the Danish Data Protection Agency (CVR-nr. 11088037-29) and the Danish National Board of Health. There was written informed consent from all patient involved in the trial, including consent to participate in the trial and consent to publish, where appropriate. Furthermore, the trial was registered at http://www.clinicaltrials.gov (ID: NCT00253071).

The participant's unique and personal identification number was submitted to the Danish National Board of Health to link with data from the Danish Psychiatric Central Register and the Danish Medical Register on Vital Statistics.

## Results

**Figure 1:**
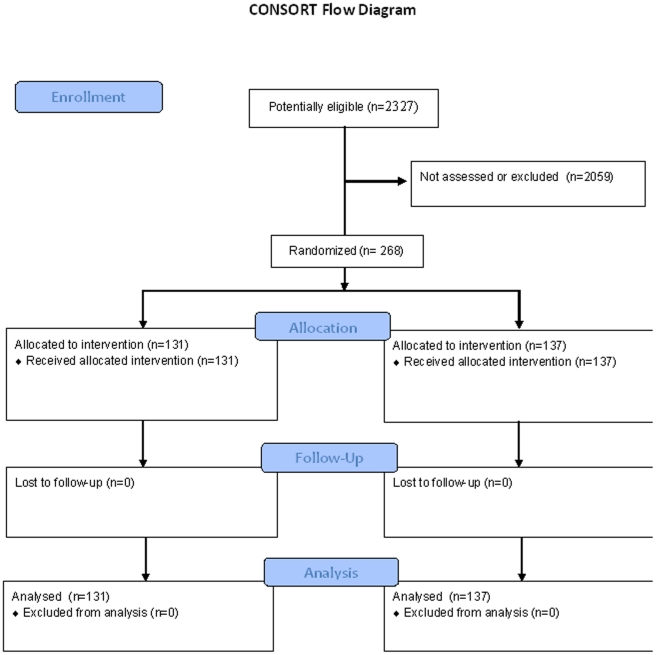
Consort flow diagram.

According to the Danish Psychiatric Central Register, 2327 patients fulfilled the inclusion criteria as they got an ICD-10 diagnosis of a single depressive episode or recurrent unipolar depression (F 32.1–F33.9) at discharge from their first, second or third psychiatric hospitalisation during the study period from December 1, 2005 to December 1, 2009. Among these potential eligible patients, 268 patients were randomised, 131 patients to treatment in the mood disorder clinic and 137 patients to standard treatment. The remaining 2059 patients were either not assessed for participation in the study (approximately 80%) or excluded due to dementia, poor Danish, any kind of commitment, or lack of informed consent. The 268 patients who participated in the trial did not differ from the 2059 potentially eligible patients regarding sex (female: 62.8% compared to 62.7%; p = 1) but were considerably younger (median 38 years (quartiles 31–53) compared to median 45 years (quartiles 31–60); p<0.001). Figure 1 is a flow diagram of patients in the trial.

The intervention in principle started at the date of discharge from the index hospitalisation as patients before discharge was allocated to receive treatment in the mood disorder clinic versus standard treatment. As national register based data on the discharge date, readmission and death were obtained for all 268 included patients and as risk time was calculated from the discharge date to readmission date, death, or end of study follow-up. No patient was lost to follow-up and no patient was excluded from the analyses.

The two intervention groups seemed reasonably well balanced regarding baseline variables, except for marital status where there was an uneven distribution of widowed patients (Table 1).

**Table 1:** Baseline socio-demographic characteristics, distribution of patients with or without previous admission before the index hospitalisation, and distribution between centers.

		The mood disorder clinic	Standard treatment
Sex	Men (%)	48 (36.4)	51 (37.2)
	Female (%)	83 (63.4)	86 (62.8)
Median age at randomisation(Quartiles)		38.4 (31.9–55.3)	38.7 (29.5–51.0)
Marital status	Married (%)	28 (22.6)	25 (19.7)
	Never been married (%)	69 (55.6)	70 (55.1)
	Divorced/separated (%)	19 (15.3)	31 (24.4)
	Widowed (%)	8 (6.5)	1 (0.8)
11 or more years of education (%)		71 (56.3)	79 (62.2)
Employment	Employed (%)	52 (42.6)	61 (49.6)
	Unemployed (%)	70 (57.4)	62 (50.4)
Number of patients with or without previous admission before index hospitalisation	Without (%)	87 (66.4)	101 (73.7)
	With (%)	44 (33.6)	36 (26.3)
Center	Hvidovre (%)	60 (45.8)	65 (47.4)
	Rigshospitalet (%)	40 (30.5)	36 (26.3)
	Amager (%)	27 (20.6)	23 (16.8)
	Others (%)	4 (3.1)	13 (9.5)

Register-based data on re-hospitalisation and death was 100% complete, i.e., available for all 268 included patients. The patients were followed to the first event, a re-admission at psychiatric hospital, or to the date of death or end of study on October 8^th^, 2010, whatever came first. Five patients died during follow-up, four patients treated in the mood disorder clinic (one due to suicide shortly after entering the clinic and three after having stopped in the clinic) and one in the standard treatment group.

**Figure 2:**
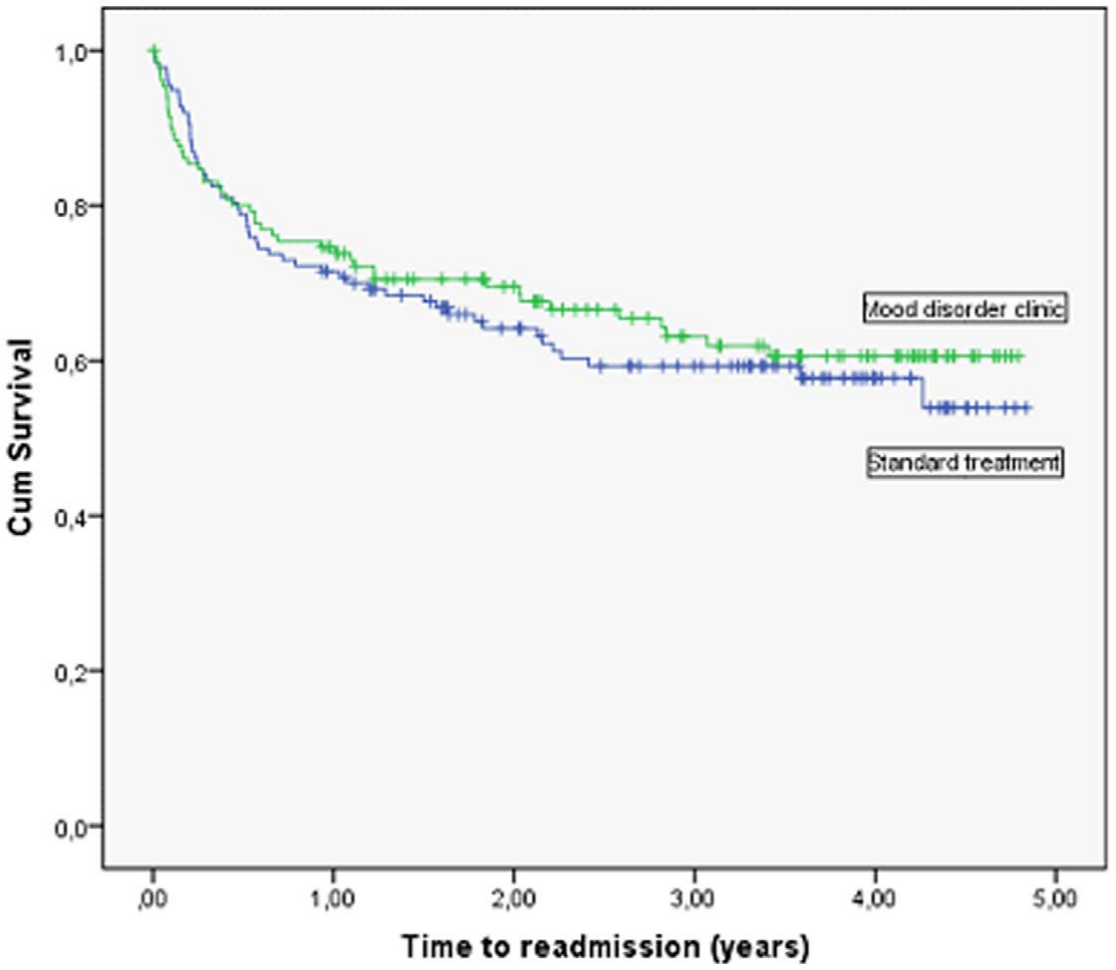
Time to readmission for patients treated in the mood disorder clinic versus standard care (all patients, N = 268).

In Figure 2, it can be seen from the Kaplan-Meier curves that patients treated in the mood disorder clinic and patients who got standard treatment did not differ significantly regarding the time to first readmission. Table 2 presents time to readmission for the two groups and a log rank test confirmed that there was no statistical significant difference between the two groups (χ^2^ = 0.3, d.f. = 1, p = 0.6; unadjusted HR 0.89, 95% CI 0.60 to 1.32; p = 0.6). When adjusted for the effect of age, sex, psychiatric centre, and number of previous psychiatric admissions in a Cox' regression model, there was no statistically significant difference between the intervention groups (HR 0.81, 95% CI 0.53 to 1.22; p = 0.3).

**Table 2:** Comparison of time to readmission for patients treated in the mood disorder clinic versus standard treatment.

		No. of patients	No. of events (readmissions, (%))	No. of events censored due to death or end of trial (%)	Mean, years of post-randomisation follow up(95% confidence interval)	Log Rank Test		
						χ^2^	d.f.	p
Treatment	The mood disorder clinic	131	47 (34.3)	84 (64.1)	3.3 (2.9–3.6)	0.3	1	0.56
	Standard treatment	137	55 (42.0)	82 (59.9)	3.1 (2.8–3.5)			

**Figure 3:**
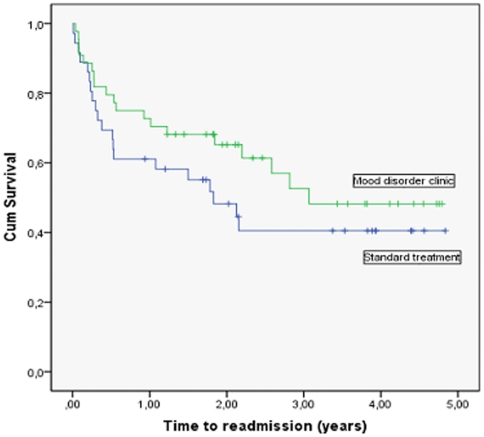
Time to readmission for patients treated in the mood disorder clinic versus standard care (only patients with two or more prior hospitalisations, N = 80).

A post-hoc explorative sub-group analysis was made in relation to the primary outcome stratifying according to the number of prior hospitalisations. Figure 3 shows Kaplan-Meier curves of time to readmission for the sub-group of patients with two or more prior hospitalisations, (n = 80, 44 in the mood disorder clinic group and 36 in the standard treatment group). There was no statistically significant differences in time to psychiatric readmission (χ^2^ = 1.4, d.f. = 1, p = 0.24; unadjusted HR 0.69, 95% CI 0.37 to 1.28; p = 0.2). Similarly, there were no significant differences in time to psychiatric readmission between the intervention groups for the 188 patients with one prior hospitalisation (unadjusted HR 0.96, 95% CI 0.58 to 1.58; p = 0.9), the 49 patients with two prior hospitalisations (unadjusted HR 0.47, 95% CI 0.19 to 1.18; p = 0.1) or the 31 patients with three prior hospitalisations (unadjusted HR 0.89, 95% CI 0.35 to 2.28; p = 0.8).

One year after discharge from the index hospitalisation, 63% of the 268 included patients fulfilled and returned the mailed questionnaire without statistically significant difference between the intervention and control groups (p = 0.6). There was no significant difference in the total score on the Major Depression Inventory (MDI) (mood disorder clinic: median 21.6 (quartiles 9.7 to 31.2) versus standard treatment: median 20.2 (quartiles 10.0 to 29.8); p = 0.7). Similarly, the prevalence of a hypomanic episode according the Mood Disorder Questionnaire (MDQ) did not differ at the one year follow-up (6.4% for patients in the mood disorder clinic versus 6.1% for patients in standard treatment; p = 0.6).

There was a significant difference in the prevalence of use a mood stabilizer (30.7% of patients treated in the mood disorder clinic compared with 17.5% in the standard treatment group; p = 0.04) but no significant difference in the prevalence of use of antidepressants (73.9% versus 80.0%, p = 0.2) or antipsychotics (12.2% versus 11.3%, p = 0.5).

Satisfaction with treatment after one year showed a highly significant difference between patients treated in the mood disorder clinic versus patients who received standard treatment (total score at VSSS-A: 131 (SD: 31.8) versus 107 (SD: 25.6); p<0.001). The overall satisfaction (items 8, 17, 18) showed a similar difference in mean score between the two groups 4.4 (SD: 1.0) versus 3.8 (SD: 1.0) in favour to the treatment in the mood disorder clinic (p<0.001).

## Discussion

The EIA trial showed no significant difference in time to psychiatric re-admission, severity of depressive symptoms, or use of pharmacological treatment (except for mood stabilizers) for patients treated for one year in a specialised outpatient mood disorder clinic versus patients offered standard outpatient treatment. Nevertheless, patients allocated to the specialised clinic were more satisfied with the care provided. There may be several explanations for these findings.

In the effort to investigate patients with early and severe depression the trial focused on patients discharged from their first, second, or third psychiatric hospitalisation. A large proportion of patients in the trial was discharged from their first psychiatric hospitalisation ever (n = 188 out of 268 (70%), Table 1). The validity of a clinical diagnosis of a single severe or moderate depressive episode have been found to be moderately high, 82.8% and 76.0% correct, respectively, according to a research based interview using the Schedules for Clinical Assessment in Neuropsychiatry (SCAN, [41]) in a prior Danish study on the validity of the diagnosis of a single depressive episode [42]. Such patients with a single severe or moderate depressive episode have a better prognosis with a lower re-admission rate than patients with several admissions [43] and we may infer from our trial that they do well with a les intensive outpatient treatment and at least they did not differ statistically significant in their re-admission rate from the mod clinic patients.

The overall re-admission rate was low in the present trial (38%) in accordance with inclusion of a large proportion of patients with a single depressive episode, who have a decreased rate of readmission. Due to the low event rate, the trial was underpowered, but we did not observe a statistically significant positive effect of the mood disorder clinic, as illustrated in Figure 1 and as reflected in the actual HR of 0.89, 95% CI 0.60 to 1.32. However, the confidence interval opens up for both a 40% relative risk reduction or a 32% relative risk increase and further trials are needed to exclude both potential benefit and harm. Register data on readmission were obtained one year after the end of the inclusion period of the trial. As we found it scientifically stringent to be blinded during the phases of data extraction and statistical analyses regarding the primary outcome throughout the study period we did not want to conduct interim analyses. Further extension of the inclusion period to more than a four year period would increase the risk of a rub-off effect from the ongoing intervention in the mood disorder clinic to intervention in standard clinical care (see later).

The patients in our trial with more severe disorders, i.e., those who had prior psychiatric admissions, seemed to have a small advantage of the interventions provided in the mood disorder clinic – but this difference was statistically insignificant (Figure 3). Findings from other studies suggest a better effect of combination therapy, pharmacological and psychological in chronic and severe depression than in less severely affected patients [44], [45]. Anyway, the present trial did not focus specifically on patients with more than one prior psychiatric admission and consequently the sample size of patients with more severe disorders was small (n = 80) resulting in decreased statistical power in this post-hoc subgroup analysis.

Comorbidity with alcohol abuse was allowed and 16% of the patients treated in the mood disorder clinic had alcohol abuse or dependency. The same distribution is expected in the control group due to the randomised design.

Bias cannot be excluded, as it was not possible to blind the physicians in the mood disorder clinic or physicians at the wards in relation to which group the patient was allocated to. Clinicians at the psychiatric wards knew before the patients were discharged from hospitalisation whether the patient was allocated to the mood disorder clinic or to standard treatment. It is possible that patients in the intervention group were discharged more early from the index hospitalisation than patients in the standard treatment group because they immediately after randomisation had a plan for outpatient treatment. However, there was no statistical significant difference in the duration of the index hospitalisation (mood disorder clinic: median duration 36.5 days, quartiles 22.0 to 75.3 versus standard treatment: median duration 42.5 days, quartiles 23.0 to 80.8, p = 0.6).

Time to (re)hospitalisation as an outcome has been criticised as reductionistic. However, it benefits from being consistently recorded and may have high face validity as admission to hospital reflects serious relapse of the illness [46]. There was no formal difference in the possibilities to re-admit patients or in practical ways of doing so between the mood disorder clinic and the psychiatric centres offering standard care. Nevertheless, it is possible that the decision to admit a patient in our trial were influenced by the treating physician's attitude toward centralised and specialised treatment although it is not obvious which directions such attitudes may cause. In the experimental group, physicians in the mood disorder clinic may be reluctant to admit a patient in order to increase the apparent benefits of the mood disorder clinic or, alternatively, to admit the patient earlier or more often as they were followed more closely. In the control group, the physicians may have been eager to admit patients to prove the advantages of the mood disorder clinic or, alternatively, be reluctant to admit the patient to prove the benefit of standard care.

It is possible that that treatment offered in standard care did not differ much from treatment offered in the mood disorder clinic. Thus, pharmacologically, only the use of mood stabilizers differed between the patient groups (30.7% of patients treated in the mood disorder clinic versus 17.5% in the standard treatment group; p = 0.04) whereas there were no significant differences in the use of antidepressants (73.9% versus 80.0%, p = 0.2) or antipsychotics (12.2% versus 11.3%, p = 0.5). Similarly, combined pharmacological and psychological intervention was also offered to a great deal of patients treated in standard care. In fact, 70.1% of patients in standard care received individual therapy and 35.0% group therapy in accordance with a growing uptake of these interventions in standard treatment. Still, even though there was no difference in the treatment response in the two groups, the patients treated in the mood disorder clinic were more satisfied with the treatment.

### Limitations

It was not possible to blind patients or treating clinicians for the intervention due to the nature of the intervention. The trial was estimating the effect of a complex intervention in a centralised mood disorder clinic versus standard treatment. The intervention consisted of a combination of many different elements and it is not be possible to differentiate the effect of the different intervention components.

The patients in the experimental group received a well-defined intervention program according to a manual. Less is known about the treatment offered the patients in the control group. It is likely that the patients in the control group received very different interventions and that these interventions varied between broad, competent and prolonged service to a much shorter and sporadic treatment offer. Furthermore, the intervention in the control group may have changed over the study period of four years to become more similar to the intervention in the experimental group because of increased focus on treatment of affective disorders possible also due to the Danish HTA report [27], the growing tendency to recommend combined pharmacological and psychological interventions for patients with unipolar disorder treated in secondary health care [47], [48], and because of a rub-off effect from the many leading local clinicians involved in the allocation of patients to the trial.

It should further be noted that according to answers on the Mood Disorder Questionnaire [49], [50] 6% to 8% of the patients might have suffered from bipolar disorder type II, not recognised by the clinicians at the local psychiatric ward. Finally, it should be recognised that the response rate to the questionnaire was disappointingly low (63%) increasing the possibility of selection bias related to the answers between the intervention groups.

### Generalisability

Pragmatic trials as the present trial are designed to measure effectiveness; that is whether an intervention works when used in usual conditions of care. To ensure applicability in the wide range of usual care settings, pragmatic trials should include all kinds of participants to whom the intervention may be offered in the real world, if its effectiveness is established. The trial included a moderately large number of patients suffering from depressive disorder with all kinds of symptoms and comorbidities and with very few exclusion criteria.

### Perspective

Overall we did not find an effect of outpatient treatment in a specialised mood disorder clinic versus standard treatment for patients early in the course of severe unipolar disorder. It seems reasonable to conclude from the trial that patients who have been hospitalised for the first time because of a single depressive episode may be treated decentralised after discharge by the general practitioner, private psychiatrist, or community psychiatric health centres. On the other hand, it cannot be excluded that patients with more severe or chronic depression with two or more previous psychiatric hospitalisations may benefit from a more specialised treatment program but this trial did not have sufficient statistical power to clarify this question. Future trials are needed to identify the group of depressive patients, if any, who might benefit from a more intensive and combined treatment program.

### What is already known on this topic

It is not known whether centralised and specialised combined pharmacological and psychological outpatient intervention in the early phase of severe unipolar depressive disorder improve prognosis compared with standard treatment.

### What this study adds

Centralised and specialised secondary outpatient care intervention following discharge from first, second or third psychiatric hospitalisation with depression resulted in no significant effects on time to rehospitalisation, severity of symptoms, or use of antidepressants compared to standard decentralised treatment, but in increased patient satisfaction.

## Supporting Information

Checklist S1 **CONSORT Checklist.**
(DOCX)

Protocol S1 **Trial Protocol.**
(PDF)

## References

[1] Kessing LV, Hansen MG, Andersen PK, Angst J (2004). The predictive effect of episodes on the risk of recurrence in depressive and bipolar disorders - a life-long perspective.. Acta Psychiatr Scand.

[2] Solomon DA, Keller MB, Leon AC, Mueller TI, Lavori PW (2000). Multiple recurrences of major depressive disorder.. Am J Psychiatry.

[3] Tohen M, Hennen J, Zarate CM, Baldessarini RJ, Strakowski SM (2000). Two-year syndromal and functional recovery in 219 cases of first-episode major affective disorder with psychotic features.. Am J Psychiatry.

[4] Kessing LV (1998). Cognitive impairment in the euthymic phase of affective disorder.. Psychol Med.

[5] Kessing LV, Nilsson FM (2003). Increased risk of developing dementia in patients with major affective disorders compared to patients with other medical illnesses.. J Affect Disord.

[6] Ownby RL, Crocco E, Acevedo A, John V, Loewenstein D (2006). Depression and risk for Alzheimer disease: systematic review, meta-analysis, and metaregression analysis.. Arch Gen Psychiatry.

[7] Geddes JR, Carney SM, Davies C, Furukawa TA, Kupfer DJ (2003). Relapse prevention with antidepressant drug treatment in depressive disorders: a systematic review.. Lancet.

[8] Jacobsen JC, Hansen JL, Storebø OJ, Simonsen E, Gluud C (2011). The effect of cognitive therapy versus ‘treatment as usual’ in patients with major depressive disorder. A systematic review of randomized clinical trials with meta-analysis and trial sequential analysis.. PLoS One.

[9] Kessing LV, Hansen MG, Andersen PK, Angst J (2004). The predictive effect of episodes on the risk of recurrence in depressive and bipolar disorders - a life-long perspective.. Acta Psychiatr Scand.

[10] Kessing LV, Hansen MG, Andersen PK (2004). Course of illness in depressive and bipolar disorders. Naturalistic study, 1994–1999.. Br J Psychiatry.

[11] Solomon DA, Keller MB, Leon AC, Mueller TI, Lavori PW (2000). Multiple recurrences of major depressive disorder.. Am J Psychiatry.

[12] Demyttenaere K, Adelin A, Patrick M, Walthere D, Katrien dB (2008). Six-month compliance with antidepressant medication in the treatment of major depressive disorder.. Int Clin Psychopharmacol.

[13] Maj M, Veltro F, Pirozzi R, Lobrace S, Magliano L (1992). Pattern of recurrence of illness after recovery from an episode of major depression: a prospective study.. Am J Psychiatry.

[14] Melfi CA, Chawla AJ, Croghan TW, Hanna MP, Kennedy S (1998). The effects of adherence to antidepressant treatment guidelines on relapse and recurrence of depression.. Arch Gen Psychiatry.

[15] Hansen HV, Kessing LV (2007). Adherence to antidepressant treatment.. Expert Rev Neurother.

[16] Von KM, Goldberg D (2001). Improving outcomes in depression.. BMJ.

[17] Badamgarav E, Weingarten SR, Henning JM, Knight K, Hasselblad V (2003). Effectiveness of disease management programs in depression: a systematic review.. Am J Psychiatry.

[18] Oxman TE, Dietrich AJ, Schulberg HC (2003). The depression care manager and mental health specialist as collaborators within primary care.. Am J Geriatr Psychiatry.

[19] Dietrich AJ, Oxman TE, Williams JW, Schulberg HC, Bruce ML (2004). Re-engineering systems for the treatment of depression in primary care: cluster randomised controlled trial.. BMJ.

[20] van Os TW, van den Brink RH, Tiemens BG, Jenner JA, van der MK (2004). Are effects of depression management training for General Practitioners on patient outcomes mediated by improvements in the process of care?. J Affect Disord.

[21] Wells K, Sherbourne C, Schoenbaum M, Ettner S, Duan N (2004). Five-year impact of quality improvement for depression: results of a group-level randomized controlled trial.. Arch Gen Psychiatry.

[22] Warner-Schmidt JL, Duman RS (2006). Hippocampal neurogenesis: opposing effects of stress and antidepressant treatment.. Hippocampus.

[23] Kessing LV, Sondergard L, Forman JL, Andersen PK (2009). Antidepressants and dementia.. J Affect Disord.

[24] Berk M, Hallam K, Lucas N, Hasty M, McNeil CA, Conus P (2007). Early intervention in bipolar disorders: opportunities and pitfalls.. Med J Aust.

[25] Zwarenstein M, Treweek S, Gagnier JJ, Altman DG, Tunis S (2008). Improving the reporting of pragmatic trials: an extension of the CONSORT statement.. BMJ.

[26] Kessing LV, Hansen HV, Christensen EM, Dam H, Gluud C (2011). The effects of centralised and specialised combined pharmacological and psychological intervention compared with decentralised and non-specialised treatment in the early course of severe unipolar and bipolar affective disorders–design of two randomised clinical trials.. Trials.

[27] Kessing LV, Hansen HV, Hougaard E, Hvenegaard A, Albæk J (2006). Preventive outpatient treatment in affective disorders..

[28] Dam H, Bendsen BB, Jakobsen K, Larsen EB, Nilsson FM (2010). [Large differences in treatment of depression between departments of psychiatry].. Ugeskr Laeger.

[29] National Board of Health (2007). Reference program for adults with unipolar depression (in Danish: Referenceprogram for unipolar depression hos voksne)..

[30] Bauer M, Whybrow PC, Angst J, Versiani M, Moller HJ (2002). World Federation of Societies of Biological Psychiatry (WFSBP) Guidelines for Biological Treatment of Unipolar Depressive Disorders, Part 1: Acute and continuation treatment of major depressive disorder.. World J Biol Psychiatry.

[31] Bauer M, Whybrow PC, Angst J, Versiani M, Moller HJ (2002). World Federation of Societies of Biological Psychiatry (WFSBP) Guidelines for Biological Treatment of Unipolar Depressive Disorders, Part 2: Maintenance treatment of major depressive disorder and treatment of chronic depressive disorders and subthreshold depressions.. World J Biol Psychiatry.

[32] Munk-Jorgensen P, Mortensen PB (1997). The Danish Psychiatric Central Register.. Dan Med Bull.

[33] Klassifikation af sygdomme. Systematisk del (1993). International Statistical Classification of Diseases and Health Related Problems..

[34] Bech P, Olsen LR (2001). [Discovering depression].. Ugeskr Laeger.

[35] Bech P, Rasmussen NA, Olsen LR, Noerholm V, Abildgaard W (2001). The sensitivity and specificity of the Major Depression Inventory, using the Present State Examination as the index of diagnostic validity.. J Affect Disord.

[36] Olsen LR, Jensen DV, Noerholm V, Martiny K, Bech P (2003). The internal and external validity of the Major Depression Inventory in measuring severity of depressive states.. Psychol Med.

[37] Hirschfeld RM, Williams JB, Spitzer RL, Calabrese JR, Flynn L (2000). Rapport DJ, Russell JM, Sachs GS, Zajecka J: Development and validation of a screening instrument for bipolar spectrum disorder: the Mood Disorder Questionnaire.. Am J Psychiatry.

[38] Hirschfeld RM, Holzer C, Calabrese JR, Weissman M, Reed M (2003). Validity of the mood disorder questionnaire: a general population study.. Am J Psychiatry.

[39] Ruggeri M, Lasalvia A, Bisoffi G, Thornicroft G, Vazquez-Barquero JL (2003). Satisfaction with mental health services among people with schizophrenia in five European sites: results from the EPSILON Study.. Schizophr Bull.

[40] Kessing LV, Hansen HV, Ruggeri M, Bech P (2006). Satisfaction with treatment among patients with depressive and bipolar disorders.. Soc Psychiatry Psychiatr Epidemiol.

[41] Wing JK, Babor T, Brugha T, Burke J, Cooper JE (1990). SCAN. Schedules for Clinical Assessment in Neuropsychiatry.. Arch Gen Psychiatry.

[42] Bock C, Bukh JD, Vinberg M, Gether U, Kessing LV (2009). Validity of the diagnosis of a single depressive episode in a case register.. Clin Pract Epidemiol Ment Health.

[43] Kessing LV, Hansen MG, Andersen PK, Angst J (2004). The predictive effect of episodes on the risk of recurrence in depressive and bipolar disorders - a life-long perspective.. Acta Psychiatr Scand.

[44] de Maat SM, Dekker J, Schoevers RA, de JF (2007). Relative efficacy of psychotherapy and combined therapy in the treatment of depression: a meta-analysis.. Eur Psychiatry.

[45] Keller MB, McCullough JP, Klein DN, Arnow B, Dunner DL (2000). A comparison of nefazodone, the cognitive behavioral-analysis system of psychotherapy, and their combination for the treatment of chronic depression.. N Engl J Med.

[46] Burns T (2009). End of the road for treatment-as-usual studies?. - Br J Psychiatry.

[47] NICE (2004). Depression: Management of depression in primary and secondary care..

[48] Danish National Board of Health (2007).

[49] Hirschfeld RM, Williams JB, Spitzer RL, Calabrese JR, Flynn L (2000). Development and validation of a screening instrument for bipolar spectrum disorder: the Mood Disorder Questionnaire.. Am J Psychiatry.

[50] Hirschfeld RM, Holzer C, Calabrese JR, Weissman M, Reed M (2003). Hazard E: Validity of the mood disorder questionnaire: a general population study.. Am J Psychiatry.

