# Supplementary material for: The Effects of Centralised and Specialised Intervention in the Early Course of Severe Unipolar Depressive Disorder: A Randomised Clinical Trial
Source: PLoS One. 2012 Mar 19;7(3):e32950. doi: 10.1371/journal.pone.0032950 (PMC3307703; doi:10.1371/journal.pone.0032950)
Supplement: Protocol S1 — Trial Protocol. (PDF) [file pone.0032950.s002.pdf]

Kessing et al. Trials 2011, 12:32
http://www.trialsjournal.com/content/12/1/32

TRIALS

STUDY PROTOCOL Open Access

The effects of centralised and specialised combined pharmacological and psychological intervention compared with decentralised and non-specialised treatment in the early course of severe unipolar and bipolar affective disorders - design of two randomised clinical trials

Lars Vedel Kessing[1*], Hanne Vibe Hansen[1], Ellen Margrethe Christensen[1], Henrik Dam[1], Christian Gluud[2], Jørn Wetterslev[2], the Early Intervention Affective Disorders (EIA) Group

Abstract

Background: In unipolar, and bipolar affective disorders, there is a high risk of relapse that increases as the number of episodes increases. Naturalistic follow-up studies suggest that the progressive development of the diseases is not prevented with the present treatment modalities. It is not known whether centralised and specialised secondary care intervention initiated early after the onset of the diseases can prevent the progression and thereby improve the prognosis.

Methods: Two randomised clinical multi-centre trials comparing a centralised and specialised outpatient intervention program consisting of combined pharmacological and psychological intervention with standard decentralised psychiatric treatment. Patients discharged from their first, second, or third hospitalisation due to a manic episode or bipolar disorder (trial 1) or to a single depressive episode or recurrent depressive disorder (trial 2) were randomised. Central randomisations for both trials were stratified for the number of hospitalisations and treatment centre. The primary outcome measure for the two trials is time to re-hospitalisation with an affective episode.

Discussion: These trials are the first to evaluate the effect of a centralised and specialised intervention in patients with early severe affective disorders. The trials used a pragmatic design comparing a specialised mood disorder clinic intervention with decentralised, non-specialised standard psychiatric treatment.

Trial Registration: ClinicalTrials.gov: NCT00253071

Background and objective

Background

Affective disorders are associated with a high risk of relapse and the risk of relapse increases as the number of previous episodes increases [1,2]. Many patients do not recover to previous psychosocial function [3,4]. A proportion of patients present with cognitive impairment also during the remitted phase [5-7], and the risk of developing dementia seems increased in the long run [8,9]. It is well documented from randomised clinical trials that the risk of a new episode in bipolar disorder can be reduced significantly by treatment with lithium or other mood stabilizers [10]. In unipolar disorder continued treatment with antidepressants significantly reduces the risk of relapse [11]. Further, the prophylactic effect of medical treatment may be enhanced by

* Correspondence: lars.vedel.kessing@regionh.dk
[1]Mood Disorder Clinic, Psychiatric Centre Copenhagen, Rigshospitalet, Copenhagen University Hospital, Copenhagen, Denmark
Full list of author information is available at the end of the article

BioMed Central

© 2011 Kessing et al; licensee BioMed Central Ltd. This is an Open Access article distributed under the terms of the Creative Commons Attribution License (http://creativecommons.org/licenses/by/2.0), which permits unrestricted use, distribution, and reproduction in any medium, provided the original work is properly cited.

psychoeducation or cognitive behavioural therapy in bipolar disorder [12-17] and by cognitive behavioural therapy in unipolar disorder [18-20]. However, results from naturalistic follow up studies suggest that the progressive development of the diseases is not prevented in clinical practice with the present treatments [21-23]. Part of the explanation may be decreased adherence with mood stabilizers and antidepressants, [24-29] but another explanations may be delayed intervention - pharmacologically and psychologically. Results from a number of studies comparing specialist first episode anti-psychosis programs versus standard treatment have shown that there are positive effects of early and sustained intervention following the first psychotic episode [30]. Within affective disorders, a number of studies have investigated the effect of health-service interventions in bipolar disorder [31-35] and recurrent depressive disorder [36-41]. However, to the best of our knowledge, no randomised clinical trial has investigated the effects of centralised and specialised combined pharmacological and psychological intervention in the early phases of unipolar or bipolar disorders. There are indirect suggestions that early intervention may improve the course and outcome in affective disorders. As summarised by Berk et al. [42], lithium for instance may be less effective in bipolar disorder as well as in unipolar disorder if not initiated early [43-45] although not all studies confirm this finding [46,47]. Further, response to cognitive behavioural therapy (CBT, [48]) and to psychoeducation [49] may be more effective when used during the early course of illnesses than after a dozen illness episodes, although a meta-analysis showed no clear evidence that the numbers of episodes moderate the effect of psychological therapy [50]. In addition, brain imaging data suggest that cognitive decline and reduction in brain volume are linked to recurrences [51] and cognitive dysfunction may increase with increasing numbers of episodes [52]. Finally, lithium and other mood stabilizers may have protective effects decreasing the tendency to cognitive decline [53-57] but existing data does not lead to any definite conclusion of a neuroprotective effect of long-term lithium therapy. Similarly, data do suggest that maintenance antidepressants prescribed for unipolar depressive disorder may have neuroprotective abilities but this is not definitive either [58,59].

It is likely that affective disorder patients are more able to benefit from medication, psychoeducation, cognitive behavioural therapy, or any other intervention if the intervention takes place early during the course of illness before a decline in psychosocial or cognitive function becomes manifest [42]. There are several difficulties in studying the effect of early intervention in affective disorders in general and bipolar disorder in specific [60]. The specificity of prodromal symptoms of the first episode is unknown and even when patients have had several episodes there is a delay of referral to treatment that prolongs time to accurate diagnosis and treatment [61]. In the present trials we focused on patients with severe affective disorders who were hospitalised for an affective episode. We only included patients after the first, second, or third time of hospitalisation. The present paper presents the background, hypotheses, design, outcomes, and statistical analyses of the two trials.

Hypotheses

Based on the literature we hypothesised that centralised and specialised outpatient intervention of patients early in the course of severe affective disorder:

1) decreases the risk of re-admission to a psychiatric hospital;
2) decreases the risk of relapse of new affective episodes;
3) increases adherence with medical treatment;
4) increases quality of life; and
5) increases satisfaction with treatment

compared with decentralised, non-specialised psychiatric outpatient treatment.

Objective

The aim of the present trials is to investigate whether centralised and specialised outpatient secondary care intervention for patients with severe affective disorder improve prognosis compared with standard psychiatric outpatient treatment in patients early in the course of mania/bipolar disorder (trial I) or depression/recurrent depressive disorder (trial II). The trials are naturalistic with very few exclusion criteria and investigate the effect among patients following psychiatric hospitalisation in The Capital Region of Denmark. When hospitalised patients were diagnosed and treated by the clinicians employed at the hospital. This pragmatic design was chosen to obtain a high generalisability of results from the trials to clinical settings regarding patients with the most severe affective disorders [62].

Methods

Study design

Patients were included from seven out of the nine psychiatric wards in The Capital Region of Denmark.

Participants and screening

According to the sample size calculation, it was planned to randomise a total of 180 patients with a manic episode/bipolar disorder (trial I) and 260 patients with a

single depressive episode/recurrent depressive disorder (trial II) to outpatient intervention in a centralised and specialised mood disorder clinic versus standard outpatient treatment. Patients in the experimental group were offered intervention in the mood disorder clinic consisting of a combination of psychopharmacological and psychological intervention and social intervention.

Randomisation

Patients were randomised to the intervention group or the control group at the end of the index hospitalisation. Randomisation was conducted centrally by the Copenhagen Trial Unit according to a computer generated allocation sequence to secure allocation concealment. In this way randomisation was independent of the professionals delivering treatment. The ratio of randomisation between the intervention and the control group was 1:1. Randomisation was stratified according to two variables: 1) psychiatric centres and 2) number of previous psychiatric hospitalisations (0, 1, or 2). The randomisations were carried out with varying block sizes unknown to the site investigators.

Inclusion and exclusion criteria

Inclusion criteria: Patients discharged from their first, second or third hospitalisation from a psychiatric ward with an ICD-10 diagnosis of a manic episode or bipolar disorder (ICD-10 code: DF30-31.9) or a single moderate or severe depressive episode or recurrent depressive disorder (ICD-10 code: DF32.1-33.9) as the primary diagnosis. Comorbidity with alcohol or substance abuse and other psychiatric disorders were allowed. The patients were diagnosed by the medical doctor at the local psychiatric ward. Age was between 18 and 70 year old. The patients were able and willing to give written and oral informed content.

Exclusion criteria: Patients with moderate or severe dementia. Patients with poor understanding of Danish language. Patients under any kind of psychiatric involuntary commitment.

Blinding

Blinding of patients and the treating clinicians was not possible as patients were randomised to the mood disorder clinic or to standard treatment. Nevertheless, statistical analyses and the writing of the scientific paper including introduction and results will be carried out before data are unblinded.

The mood disorder clinic

Patients in the intervention group were treated in a specialised outpatient mood disorder clinic at Psychiatric Centre Copenhagen, Rigshospitalet, Copenhagen University Hospital. The clinic was established September 2004 in parallel to the work and publication of a Health Technology Assessment (HTA) report on outpatient treatment in severe affective disorders [63], chaired by the last (LVK) and first author (HVH)). The HTA report examined the following three key aspects of outpatient treatment of patients with major affective disorder:

- Prophylactic pharmacotherapy.
- Prophylactic combination therapy (pharmacotherapy and psychological intervention/psychosocial support).
- Doctor-patient cooperation expressed as the degree of adherence to the internationally recommended treatment guidelines.

It was recommended that the current organisation of outpatient treatment of patients with depressive or bipolar affective disorder should be supplemented with 5-10 specialised clinics in Denmark and that the clinics should:

1. be a supplement to the present decentralised treatment;
2. provide treatment of the highest professional standard;
3. offer both pharmacotherapy and psychological intervention;
4. regularly perform quality assurance and quality development of the interventions; and
5. provide education and perform research in diagnosis and treatment of affective disorder.

The staff in the outpatient mood disorder clinic at Rigshospitalet, Copenhagen University Hospital consists of full time specialists in psychiatry with specific clinical experience and knowledge on diagnosis and treatment of affective disorders as well as certified psychologists, nurses and a social worker with experience within affective disorders. The staff in the mood disorder clinic did not provide treatment to any of the patients in the standard treatment group.

Intervention group

During the first year following establishment of the clinic a detailed intervention program including manuals for psychological group interventions (psychoeducation and cognitive behavioural therapy) was developed, tested, and revised in a pilot phase with inclusion of approximately 30 patients. The final combined pharmacological and non-pharmacological intervention program was as following. Separate intervention programs are provided for patients with bipolar disorder and patients for unipolar disorder lasting two years for patients with bipolar disorder (trial I) and one year for patients with

unipolar disorder (trial II)(a minor proportion of patients who did not achieve remission during these periods were offered further individual treatment). According to the protocol, the physician evaluates all patients in the clinic less than two weeks after discharge from the local psychiatric ward. Although most patients improve during hospitalisation for affective disorders it is well known that they do still suffer from affective symptoms at discharge from hospitals in the Copenhagen area [64]. Prior course of illness and effect of treatment is carefully recorded and diagnosis and treatment plans are re-evaluated and current pharmacological treatment adjusted in accordance with clinical status and the national [65,66] and international guidelines [67-71]. Patients with a unipolar depressive disorder were offered acute and prophylactic treatment with ssri's/dual action drugs depending on individual effects and side effects. In case of poor response, switch was made to nortriptyline and if necessary, subsequently in combination with lithium. The main mood stabilisers for bipolar disorder were lithium, lamotrigine and valproate that for some patients were combined with atypical antipsychotics (mainly olanzapine, quetiapine, aripiprazole) or antidepressants for shorter time periods. Decisions on patient's individual pharmacotherapy were made by the individual physician in accordance with the other specialists in psychiatry and other providers at weekly staff conferences. Prescription of medication and adherence to medication will be characterised using the national medication register of all medication purchased at pharmacies in Denmark. Subsequently, the physician follows the patients with regular appointments depending on their clinical status and needs. In addition, patients participate in three different sequential group sessions weekly. All sessions are carried out in accordance with the manuals although individualised according to characteristics and needs of patients in the group. The first group is a settling-in group for patients just discharged from hospitalisation and with focus on the current clinical status, beliefs, and experiences in relation to the recent hospitalisation. Patients stay in this group until they are clinically stable and in at least partly remitted from affective symptoms (Hamilton Depression Score-17 items <14 and Young Rating Mania Scale <14), i.e., typically for some months up to half a year. When stable, the patients join a second and intermediary group consisting of either group psychoeducation or cognitive behavioural therapy. The type of group therapy is decided by the clinician and the patient in collaboration. The group sessions consist of 11/2 hours intervention every week for 12 consecutive weeks. In both groups, focus is on knowledge and acceptance of suffering from an affective disorder (bipolar or unipolar), identifying affective symptoms from normal reactions, personal identity in relation to suffering from an affective disorder, risk situations, stress management, the need for sustained pharmacological maintenance treatment, adverse events due to treatment, and identification of individual prior early warning signs of upcoming affective episodes. In addition, the cognitive behavioural therapy group sessions focus on inter-individual conflicts and cognitive distortions in identity and behaviour. Finally, the patients join a 3-6 months training discharge group that is a preparation for re-referral to the initially referring physician with the aim of identifying individual early warning signals *prospectively* in practice and training of how to change upcoming personal conflicts and cognitive distortions.

Six to eight patients and two therapists (psychiatrist, psychologist, or nurse) participate in each group. In the cognitive behavioural groups, at least one therapist has a formal education in cognitive behavioural therapy. Adherence to group treatment programmes is recorded. Parallel to these sessions, relatives to patients with bipolar disorder were offered a manual based psychoeducative group course consisting of 2-hours sessions weekly for six weeks.

The physician directs and co-ordinates treatments and makes decisions on discharge, in accordance with other health-care providers.

Control group

The control group of patients were offered standard outpatient care consisting of the standard mental health service routines in The Capital Region of Denmark which is rather similar across the geographical catchments areas associated with the seven wards, i.e., treatment at the general practitioner, a private psychiatrist, or at the community mental health centre. Participation in the trials had no influence on the treatment offered to these patients. Compared with the intervention in the mood disorder clinic, psychopharmacological treatment is likely to be more based on the preferences of the individual medical physician than on national and international guidelines. Prescription of medication will be characterised using the national medication register of all medication purchased at pharmacies in Denmark. Psychosocial treatment elements like psychoeducation and cognitive behavioural therapy, and contact with family is in general provided infrequently and in a less intensive, non-systematic way and only for a minority of the control patients.

Outcome assessments

Patients in the experimental intervention group and in the control group had been hospitalised to the same psychiatric departments prior to randomisation and would be readmitted to the same department if re-hospitalisation would be needed following randomisation.

The primary outcome is time from randomisation to first re-admission to a psychiatric ward with an affective episode. Data on re-hospitalisation will be obtained one, two, and five years following randomisation from the nation-wide Danish Psychiatric Central Register that contains data on all inpatients and outpatients contacts to all psychiatric hospital based services in Denmark [72]. Since 1 January 1994 the ICD-10 has been in use in the register [73].

The secondary outcome measures are 1) the development of a depressive or manic/mixed episode of at least moderate severity - together and separately, one and two years after randomisation and 2) adherence to maintenance antidepressant and mood stabilizing treatments one and two years after randomisation. These outcomes were assessed using questionnaires that were send to all participants one and two years following randomisation: the Major Depression Inventory (MDI) [74-76] and the Mood Disorder Questionnaire (MDQ) [77,78]. A depressive episode (of at least moderate severity) is defined as the presence of two or three depressive core symptoms and four to seven accompanying symptoms on the MDI. A manic or mixed episode is defined as a score of seven or more on the MDQ. The Antidepressant Compliance Questionnaire and the Mood stabilizer Compliance Questionnaire were used for measuring compliance with treatment modified after Demyttenaere [79,80].

The tertiary outcome are satisfaction with the intervention one and two years after randomisation estimated by the WHO (Five) well-being index [81,82] and the Verona Satisfaction Scale-Affective Disorder (VSS-A) [83]. Death due to suicide will be identified using register data from the Danish Medical Register on Vital Statistics [84].

Psychiatric outpatient contact and use of medication will be analysed for the intervention and control groups using register based data. Thus, we will have complete (register based) data on re-hospitalisation, medication and suicide whereas questionnaire based data will be missing for some patients who drop out of the trials.

Sample size calculation

Anticipating a hazard ratio (HR) of 0.65 in the comparison of the intervention group with the control group on the primary outcome, a two-sided risk of type 1 error, α, of 0.05, a type 2 error risk, β, of 20%, and equal group size, the sample size (N) for trial I was calculated to N = 176 under the further assumption of a median time to re-hospitalisation of approximately 6 months in the control group, an inclusion period on 36 months, and a follow up period of 12 months. The sample size in trial II was based on similar assumptions (see Figure 1). However, patients with unipolar depressive disorder are more prevalent than patients with mania or bipolar disorder. Hence, we expected to be able to randomise more patients with unipolar depressive disorder. If we finally accrued a higher sample size in trial II compared to trial I, then the power for detection of a HR of 0.65 would become higher than 80%. The required sample sizes were calculated with the prospect of using a log rank test-statistic to test the null-hypothesis of no difference in time from randomisation to the primary outcome of re-admission to psychiatric hospital.

The assumption of time to re-hospitalisation was based on national hospital data showing that half of all patients hospitalised with a diagnosis of depression or mania/bipolar disorder are re-hospitalised within a few months [85]. The assumption that the proportion of re-admission would be 35% decreased in the intervention group compared with the intervention group was based on findings of the effect of psychoeducation in bipolar disorder [86] and further on presumptions of positive effects of the early contact to patients during the vulnerable period following hospital discharge (less than two weeks) and a low drop out in the intervention group as experienced during the pilot phase of the trials.

Statistical analyses

Separate statistical analyses will be made for patients with bipolar disorder and patients with unipolar disorder. The statistical analyses will be made as 'intention-to-treat' analyses. Regarding the primary outcome, time to the first re-hospitalisation will be estimated in Kaplan-Meier plots. The difference in cumulated prevalence of re-hospitalisation in the intervention and in the control group will be tested in log-rank tests. The absolute and relative hazard risk reductions and the corresponding 95% confidence intervals will be calculated. In addition, hazard ratios adjusted for age, sex, psychiatric centre, and number of previous admissions will be calculated in Cox' regression models.

Analyses comparing participants and eligible non-participants in the trials will be carried out using data from the Danish Central Psychiatric Register to identify any difference between these groups regarding sex, age, primary diagnosis, diagnoses of comorbidities, and number of previous admissions to psychiatric hospitals.

Ethical considerations

The trials are approved by the Danish Research Ethical Committee (KF 01 272130) and the Danish Data Protection Agency (CVR-nr. 11-88-37-29). We have written informed consent from any patients involved in the trials, including consent to participate in the trials and consent to publish, where appropriate. Further, the trials are registered at http://www.clinicaltrials.gov (ID: NCT00253071). The trial participant's unique and

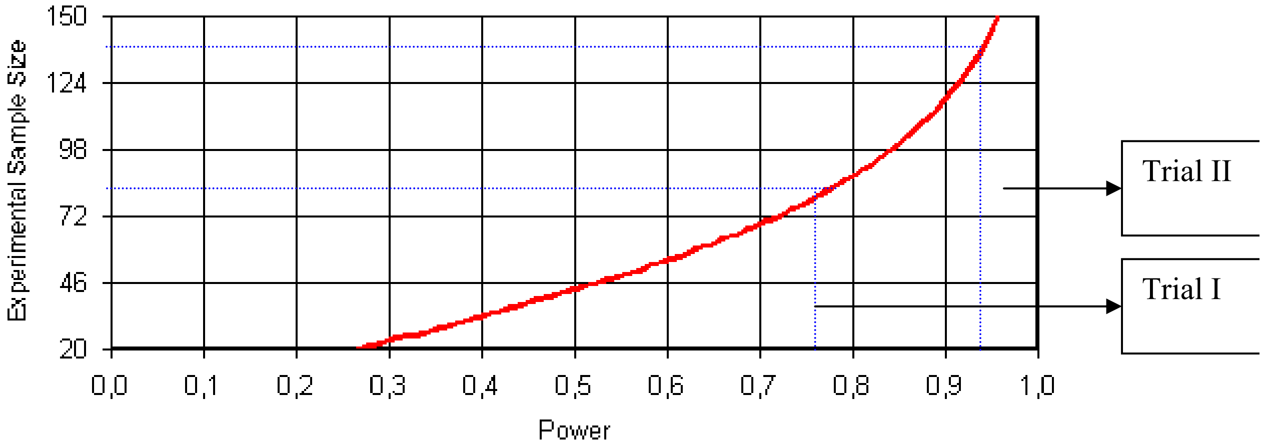

Figure 1 Anticipated statistical power estimations in Trials I and II. The anticipated power in the trials on the x-axis as a function of the sample size of the trials on the y-axis based on α = 0.05 and an anticipated hazard ratio of 0.65 and 6 month median time from randomisation to re-admission to hospital in the control group, 48 month inclusion period, and at least a 12 month follow up of all patients.

personal identification number will be submitted to the Danish National Board of Health in order to link to data from the Danish Central Psychiatric Register, the Medicinal Product Statistics, and the Danish Medical Register on Vital Statistics to the study data.

Results

Current trial status

Recruitment of bipolar patients was somewhat slower than expected, so the recruitment period had to be extended one year further than the originally planned three-year inclusion period for both patient groups. Thus, patient enrolment started in December 2005 and closed in December 2009, resulting in enrolment and randomisation of a total of 426 participants, 158 with bipolar disorder and 268 with unipolar disorder. Accordingly, we did not reach the planned number of 176 patients with bipolar affective disorder. Among unipolar patients, 63.1% were women and the median age was 38.6 years (quartiles: 31.1 to 53.4). Among bipolar patients, 54.4% were women and the median age was 35.6 years (quartiles: 27.7 to 47.1).

Power calculations for trials I and II

In the bipolar disorder trial I, the number of 158 recruited patients results in a power of 76% (power = 1-β) for the detection or rejection of a HR of 0.65. In the unipolar disorder trial II, the number of 268 recruited patients results in a power of 93% for the detection or rejection of a HR of 0.65. The power in trial I would be 60% and the power in trial II 82% to detect or reject a HR of 0.70. The power in trial I would be 42% and the power in trial II 63% to detect or reject a HR of 0.75. The statistical power in the two trials is calculated with the prospect of using a log rank test-statistic to test the null-hypothesis of no difference in time from randomisation to the primary outcome.

Discussion

Limitations

It is an inherent limitation of the trials designs that it is not possible to blind patients and treating clinicians for intervention due to the nature of the intervention (specialised out-patient intervention versus standard treatment). This may limit the inferences based on these trials. The trials aim to investigate the effect of specialised and centralised secondary care intervention early in the course of severe affective disorders, i.e., in patients discharged from their first, second, or third hospitalisation. It is evident that some of these patients may have experienced prior affective episodes less severe than those resulting in hospitalisation and there may have been a delay in seeking help and diagnosis of the mood disorder [45,46] as reflected in the relatively high median age (especially for bipolar patients). Nevertheless, randomisation should take care of this potential confounder in our analyses.

Further, it should be noted that the study is estimating the effect of a complex intervention in a centralised and specialised mood disorder clinic. The intervention consists of a combination of many different elements and we suspect it will be impossible to differentiate the effect of the different intervention components consisting of medical treatments, psychoeducation, cognitive behavioural intervention, and social support.

The patients in the experimental intervention group received a well-defined intervention program according to a manual. Little is known about the treatment offered to the patients in the control group. It is likely that the patients in the control group received very different

intervention offers and that these interventions varied between a broad, competent and prolonged service to a much shorter and sporadic offer of treatment. Furthermore, the intervention in the control group may change over time to become similar to the intervention in the experimental group because of increased focus on treatment of affective disorders (possibly also due to the Danish HTA report [63]), the growing evidence of the effect of combined intervention (pharmacologically and psychologically) and because of a rub-off effect from the many leading local clinicians involved in the allocation of patients to the trials.

Even though there were very few exclusion criteria, it is likely that patients who accept randomisation may be a selected group and that patients with the most severe illness, with comorbidity, e.g., substance abuse are under-represented. Furthermore, the current design may introduce attrition bias as the more severely ill and patients with comorbid substance use problems and other comorbidity may be more able and willing to attend locally tailored treatment rather than centralised treatment. Participants and eligible non-participants will be compared using register-based variables to evaluate whether participants in the trials are representative of patients with affective disorders, discharged form their first, second or third hospitalisation.

Advantages

Central randomisation protect against selection bias in the randomisation process. Further, it is a major advantage that the primary outcome measure, i.e., first re-admission to a psychiatric ward can be obtained for all included patients in the trials based on data from a nation-wide health register data [72]. Data on outpatient pharmacological treatment will be obtained from the Medicinal Product Statistics that contains data on all prescribed medication purchased nationwide at pharmacies from January 1, 1995 and onwards [87].

Generalisability

Pragmatic trials as the present trials are designed to measure effectiveness; that is whether an intervention works when used in usual conditions of care. To ensure applicability in a wide range of usual care settings, pragmatic trials should include all kinds of participants to whom the intervention may be offered in the real world, if its effectiveness is established [62]. A total of seven psychiatric centres participated in our trials out of the 9 centres that offer acute hospitalisation in The Capital Region of Denmark. The two non-participating centres do not differ noticeably in patient population of affective disorders or treatment services from the seven centres. The trials include a moderately large number of patients and have a naturalistic design with inclusion of patients suffering from severe affective disorders with all kinds of symptoms and comorbidities and with very few exclusion criteria. Thus, patients with depressive, manic or mixed index episodes, early and late onset, with or without psychosis and comorbidity, such as personality disorders, alcohol or substance abuse, etc, as well as patients with poor or good treatment outcome during hospitalisation were included. It should be noted that participants were included in the trials based on the diagnoses established by the treating medical doctors employed at the psychiatric hospitals to obtain a high generalisability of results from the trials to clinical settings regarding patients with the most severe affective disorders. Including only patients who fulfil diagnostic criteria for an affective disorder according to a research-based interview (such as, e.g., the SCAN interview) in the trials would not mimic the naturalistic clinical setting to which we wanted the results to be generalisable. Nevertheless, the validity of diagnoses made by physicians at discharge from psychiatric hospitals in Denmark is reasonably high compared to research-based diagnostic interviews with a probability of a correct diagnosis for bipolar disorder of 94% [88] and with a probability of a correct diagnosis for unipolar disorder of 83% [89].

Hospitalisation as an outcome has been criticised as reductionistic. However, it benefits from being consistently recorded and has high face validity as admission to hospital reflects a serious relapse of the illness [90]. Nevertheless, it is possible that the decision to admit a patient in our trials were influenced by the treating physicians' attitude toward centralised and specialised treatment although it is not evident that such attitudes may cause bias. In the experimental group, physicians in the mood disorder clinic may be reluctant to admit a patient in order to increase the apparent benefits of the mood disorder clinic or, in contrast, admit the patient earlier and more often as these are followed more closely, which will make the experimental intervention look worse. In the control group, the physicians may be eager to admit a patient to prove the advantages of the mood disorder clinic or, in contrast, be reluctant to admit the patient to prove the benefits of standard care. We are able to estimate the effect of such potential bias by comparing diagnoses on severity of the illness leading to hospitalisation (current depressive episode mild, moderate or severe, current episode hypomanic or manic; with or without psychotic symptoms) given by the inpatient physicians at discharge from the outcome hospitalisation for the intervention and the control groups, as these are recorded in the Danish Psychiatric Central Register. The physicians at the psychiatric centre give diagnoses independently of the referring outpatient physician. In addition, it is not clear that the potential effect of such attitudes differs in the experimental trial setting and the non-experimental everyday clinic. It should be

realised that the rate of (re-) hospitalisation in the trials should be considered in relation to other outcome measures such as the proportion with relapse and suicide in the two groups of the trials. Notably, researchers did not assess patients during the follow-up as the primary outcome as well as one tertiary (suicide) outcome was based on register data and other outcomes were based on a questionnaire mailed to the participants. Using this simple approach, the research team did not interact with the patients in the trials in any way, i.e., the research team had no effect on inclusion of participants into the trials, delivered treatment or drop-out from treatment, in contrast to what is the case in trials with extensive and repeated assessment by researchers. Our trials are thus designed to closely imitate the clinical situation and results from the trials may be generalised to patients discharged from early admissions with a manic episode/bipolar disorder or a single moderate to severe depressive episode or recurrent depressive disorder, respectively, and the results should be generalisable into the everyday clinic.

The downside of this simple approach is that the secondary and most tertiary outcomes are based on patient's self-assessment that may have a decreased validity, especially during manic episodes. We used the Mood Disorder Questionnaire (MDQ) to assess the development of a manic/mixed episode of at least moderate severity although it is critical whether this is a reliable approach.

The health care system in The Capital Region of Copenhagen is similar to the health care system elsewhere in Denmark and to the majorities of Western countries with a large primary health care system of general practitioners and private specialists and a smaller secondary health care sector including hospitals and community psychiatric centres. Thus, we believe that the results of the trials should be generalisable to most Western communities.

Perspectives

The trials will evaluate the effect of a centralised and specialised secondary care intervention early in the course of severe affective disorder. Findings of a positive effect should be interpreted with caution due to the lack of blinding possibilities and further in combination with results from economical evaluations. Nevertheless, providing evidence of a benefit of intensive outpatient interventions for patients early in the diseases of bipolar or depressive disorder may have great influence on future treatment for the patient groups. The perspective is to be able to prevent some of the negative progression and consequences of major affective disorders.

Acknowledgements

The study is supported by the Lundbeck Foundation and by the Research Foundation of the Hovedstadens Sygehusfællesskab (The Capital Hospital Corporation).

Early Intervention Affective Disorders (EIA) Group***

***Psychiatric Centre Hvidovre; Psychiatric Centre Copenhagen, Rigshospitalet; Psychiatric Centre Amager; Psychiatric Centre Frederiksberg, Psychiatric Centre Copenhagen, Bispebjerg; Psychiatric Centre, Gentofte; and Psychiatric Centre Hillerød, Copenhagen University Hospital, Denmark.

*** Members of the EIA Group also encompass Klaus D, Ejnar B Larsen, Psychiatric Centre Hvidovre; Martin B Jørgensen, Maj Vinberg, Rikke Engel, Psychiatric Centre Copenhagen, Rigshospitalet; Flemming M Nilsson, Nana Hengstenberg, Psychiatric Centre Amager; Birgitte B Bendsen, Psychiatric Centre Frederiksberg; Hans M Jensen, Psychiatric Centre Copenhagen, Bispebjerg; Rie L Mikkelsen, Psychiatric Centre Gentofte; Birgit Straasø, Jens Abraham, Psychiatric Centre Hillerød.

Author details

[1]Mood Disorder Clinic, Psychiatric Centre Copenhagen, Rigshospitalet, Copenhagen University Hospital, Copenhagen, Denmark. [2]Copenhagen Trial Unit (CTU), Centre for Clinical Intervention Research, Rigshospitalet, Copenhagen University Hospital, Copenhagen, Denmark.

Authors' contributions

LVK and HVH conceived the trials and authored the first draft of the trial protocols and this report. HD, CG, and JW have been revising and optimising the trial protocols and the article. The Copenhagen Trial Unit conducted the centralised randomisation. LVK and EMC have been responsible for treatment of the patients in the experimental intervention group. Seven psychiatric centres in the greater Region Copenhagen are involved in the trials specifically in the recruitment of patients and randomisation and are represented by on or two senior consultants in psychiatry in the Early Intervention Affective Disorder (EIA) Group. All authors read and approved the final manuscript.

Competing interests

Lars Vedel Kessing has been a consultant for Bristol-Myers Squibb, Eli Lilly, Lundbeck, AstraZenica, Pfizer, Wyeth, and Servier. Ellen Margrethe Christensen has been a consultant for Bristol-Myers Squibb, AstraZeneca, Eli Lilly, MSD, and Servier. Henrik Dam, Christian Gluud, and Jørn Wetterslev have no competing interests.

Received: 27 September 2010 Accepted: 3 February 2011
Published: 3 February 2011

References

1. Kessing LV, Hansen MG, Andersen PK, Angst J: **The predictive effect of episodes on the risk of recurrence in depressive and bipolar disorders - a life-long perspective.** *Acta Psychiatr Scand* 2004, **109(5)**:339-344.
2. Solomon DA, Keller MB, Leon AC, *et al*: **Multiple recurrences of major depressive disorder.** *Am J Psychiatry* 2000, **157(2)**:229-233.
3. Strakowski SM, Keck PE Jr, Sax KW, McElroy SL, Hawkins JM: **Twelve-month outcome of patients with DSM-III-R schizoaffective disorder: comparisons to matched patients with bipolar disorder.** *Schizophr Res* 1999, **35(2)**:167-174.
4. Tohen M, Hennen J, Zarate CM Jr, *et al*: **Two-year syndromal and functional recovery in 219 cases of first-episode major affective disorder with psychotic features.** *Am J Psychiatry* 2000, **157(2)**:220-228.
5. Kessing LV: **Cognitive impairment in the euthymic phase of affective disorder.** *Psychol Med* 1998, **28(5)**:1027-1038.
6. Torres IJ, Boudreau VG, Yatham LN: **Neuropsychological functioning in euthymic bipolar disorder: a meta-analysis.** *Acta Psychiatr Scand Suppl* 2007, , **434**: 17-26.
7. Hasselbalch BJ, Knorr U, Kessing LV: **Cognitive impairment in the remitted state of unipolar depressive disorder: A systematic review.** *Journal of Affective Disorders* 2010.
8. Kessing LV, Nilsson FM: **Increased risk of developing dementia in patients with major affective disorders compared to patients with other medical illnesses.** *J Affect Disord* 2003, **73(3)**:261-269.

9. Ownby RL, Crocco E, Acevedo A, John V, Loewenstein D: Depression and risk for Alzheimer disease: systematic review, meta-analysis, and metaregression analysis. *Arch Gen Psychiatry* 2006, 63(5):530-538.
10. Goodwin GM, Geddes JR: Latest maintenance data on lithium in bipolar disorder. *Eur Neuropsychopharmacol* 2003, 13(Suppl 2):S51-5-S51-S55.
11. Geddes JR, Carney SM, Davies C, *et al*: Relapse prevention with antidepressant drug treatment in depressive disorders: a systematic review. *Lancet* 2003, 361(9358):653-661.
12. Colom F, Vieta E, Martinez-Aran A, *et al*: A randomized trial on the efficacy of group psychoeducation in the prophylaxis of recurrences in bipolar patients whose disease is in remission. *Arch Gen Psychiatry* 2003, 60(4):402-407.
13. Huxley NA, Parikh SV, Baldessarini RJ: Effectiveness of psychosocial treatments in bipolar disorder: state of the evidence. *Harv Rev Psychiatry* 2000, 8(3):126-140.
14. Lam DH, Watkins ER, Hayward P, *et al*: A randomized controlled study of cognitive therapy for relapse prevention for bipolar affective disorder: outcome of the first year. *Arch Gen Psychiatry* 2003, 60(2):145-152.
15. Maj M, Veltro F, Pirozzi R, Lobrace S, Magliano L: Pattern of recurrence of illness after recovery from an episode of major depression: a prospective study. *Am J Psychiatry* 1992, 149(6):795-800.
16. Miklowitz DJ, George EL, Richards JA, Simoneau TL, Suddath RL: A randomized study of family-focused psychoeducation and pharmacotherapy in the outpatient management of bipolar disorder. *Arch Gen Psychiatry* 2003, 60(9):904-912.
17. Morriss RK, Faizal MA, Jones AP, Williamson PR, Bolton C, McCarthy JP: Interventions for helping people recognise early signs of recurrence in bipolar disorder. *Cochrane Database Syst Rev* 2007, , 1: CD004854.
18. Paykel ES, Scott J, Teasdale JD, *et al*: Prevention of relapse in residual depression by cognitive therapy: a controlled trial. *Arch Gen Psychiatry* 1999, 56(9):829-835.
19. Segal Z, Vincent P, Levitt A: Efficacy of combined, sequential and crossover psychotherapy and pharmacotherapy in improving outcomes in depression. *J Psychiatry Neurosci* 2002, 27(4):281-290.
20. Teasdale JD, Segal ZV, Williams JM, Ridgeway VA, Soulsby JM, Lau MA: Prevention of relapse/recurrence in major depression by mindfulness-based cognitive therapy. *J Consult Clin Psychol* 2000, 68(4):615-623.
21. Solomon DA, Keller MB, Leon AC, *et al*: Multiple recurrences of major depressive disorder. *Am J Psychiatry* 2000, 157(2):229-233.
22. Kessing LV, Hansen MG, Andersen PK: Course of illness in depressive and bipolar disorders. Naturalistic study, 1994-1999. *Br J Psychiatry* 2004, 185:372-377.
23. Kessing LV, Hansen MG, Andersen PK, Angst J: The predictive effect of episodes on the risk of recurrence in depressive and bipolar disorders - a life-long perspective. *Acta Psychiatr Scand* 2004, 109(5):339-344.
24. Vestergaard P, Schou M: Prospective studies on a lithium cohort. 1. General features. *Acta Psychiatr Scand* 1988, 78(4):421-426.
25. Maj M, Veltro F, Pirozzi R, Lobrace S, Magliano L: Pattern of recurrence of illness after recovery from an episode of major depression: a prospective study. *Am J Psychiatry* 1992, 149(6):795-800.
26. Melfi CA, Chawla AJ, Croghan TW, Hanna MP, Kennedy S, Sredl K: The effects of adherence to antidepressant treatment guidelines on relapse and recurrence of depression. *Arch Gen Psychiatry* 1998, 55(12):1128-1132.
27. Demyttenaere K, Adelin A, Patrick M, Walthere D, Katrien dB, Michele S: Six-month compliance with antidepressant medication in the treatment of major depressive disorder. *Int Clin Psychopharmacol* 2008, 23(1):36-42.
28. Hansen HV, Kessing LV: Adherence to antidepressant treatment. *Expert Rev Neurother* 2007, 7(1):57-62.
29. Kessing LV, Sondergard L, Kvist K, Andersen PK: Adherence to lithium in naturalistic settings: results from a nationwide pharmacoepidemiological study. *Bipolar Disord* 2007, 9(7):730-736.
30. varez-Jimenez M, Parker AG, Hetrick SE, McGorry PD, Gleeson JF: Preventing the Second Episode: A Systematic Review and Meta-analysis of Psychosocial and Pharmacological Trials in First-Episode psychosis. *Schizophr Bull* 2009.
31. Bauer MS, Williford WO, Dawson EE, *et al*: Principles of effectiveness trials and their implementation in VA Cooperative Study #430: 'Reducing the efficacy-effectiveness gap in bipolar disorder'. *J Affec Disord* 2001, 67(1-3):61-78.
32. Bauer MS, McBride L, Williford WO, *et al*: Collaborative care for bipolar disorder: Part II. Impact on clinical outcome, function, and costs. *Psychiatr Serv* 2006, 57(7):937-945.
33. Bauer MS, McBride L, Williford WO, *et al*: Collaborative care for bipolar disorder: part I. Intervention and implementation in a randomized effectiveness trial. *Psychiatr Serv* 2006, 57(7):927-936.
34. Bauer MS, Biswas K, Kilbourne AM: Enhancing multiyear guideline concordance for bipolar disorder through collaborative care. *Am J Psychiatry* 2009, 166(11):1244-1250.
35. Simon GE, Ludman EJ, Bauer MS, Unutzer J, Operskalski B: Long-term effectiveness and cost of a systematic care program for bipolar disorder. *Arch Gen Psychiatry* 2006, 63(5):500-508.
36. Wells K, Sherbourne C, Schoenbaum M, *et al*: Five-year impact of quality improvement for depression: results of a group-level randomized controlled trial. *Arch Gen Psychiatry* 2004, 61(4):378-386.
37. van Os TW, van den Brink RH, Tiemens BG, Jenner JA, Van der MK, Ormel J: Are effects of depression management training for General Practitioners on patient outcomes mediated by improvements in the process of care? *J Affect Disord* 2004, 80(2-3):173-179.
38. Dietrich AJ, Oxman TE, Williams JW Jr, *et al*: Re-engineering systems for the treatment of depression in primary care: cluster randomised controlled trial. *BMJ* 2004, 329(7466):602.
39. Badamgarav E, Weingarten SR, Henning JM, *et al*: Effectiveness of disease management programs in depression: a systematic review. *Am J Psychiatry* 2003, 160(12):2080-2090.
40. Oxman TE, Dietrich AJ, Schulberg HC: The depression care manager and mental health specialist as collaborators within primary care. *Am J Geriatr Psychiatry* 2003, 11(5):507-516.
41. Von Korff M, Goldberg D: Improving outcomes in depression. *Br med J* 2001, 323:7319-7949.
42. Berk M, Hallam K, Malhi GS, *et al*: Evidence and implications for early intervention in bipolar disorder. *J Ment Health* 2010, 19(2):113-126.
43. Franchini L, Zanardi R, Smeraldi E, Gasperini M: Early onset of lithium prophylaxis as a predictor of good long-term outcome. *Eur Arch Psychiatry Clin Neurosci* 1999, 249(5):227-230.
44. Swann AC, Bowden CL, Calabrese JR, Dilsaver SC, Morris DD: Differential effect of number of previous episodes of affective disorder on response to lithium or divalproex in acute mania. *Am J Psychiatry* 1999, 156(8):1264-1266.
45. Post RM, Leverich GS, Altshuler LL, *et al*: An overview of recent findings of the Stanley Foundation Bipolar Network (Part I). *Bipolar Disord* 2003, 5(5):310-319.
46. Baethge C, Smolka MN, Gruschka P, *et al*: Does prophylaxis-delay in bipolar disorder influence outcome? Results from a long-term study of 147 patients. *Acta Psychiatr Scand* 2003, 107(4):260-267.
47. Baldessarini RJ, Tondo L, Hennen J: Treatment-latency and previous episodes: relationships to pretreatment morbidity and response to maintenance treatment in bipolar I and II disorders. *Bipolar Disord* 2003, 5(3):169-179.
48. Scott J, Paykel E, Morriss R, *et al*: Cognitive-behavioural therapy for severe and recurrent bipolar disorders: randomised controlled trial. *Br J Psychiatry* 2006, 188:313-320.
49. Vieta E, Pacchiarotti I, Valenti M, Berk L, Scott J, Colom F: A critical update on psychological interventions for bipolar disorders. *Curr Psychiatry Rep* 2009, 11(6):494-502.
50. Lam DH, Burbeck R, Wright K, Pilling S: Psychological therapies in bipolar disorder: the effect of illness history on relapse prevention - a systematic review. *Bipolar Disord* 2009, 11(5):474-482.
51. Berk M, Hallam K, Malhi GS, *et al*: Evidence and implications for early intervention in bipolar disorder. *J Ment Health* 2010, 19(2):113-126.
52. Robinson LJ, Ferrier IN: Evolution of cognitive impairment in bipolar disorder: a systematic review of cross-sectional evidence. *Bipolar Disord* 2006, 8(2):103-116.
53. Lagace DC, Eisch AJ: Mood-stabilizing drugs: are their neuroprotective aspects clinically relevant? *Psychiatr Clin North Am* 2005, 28(2):399-414.
54. Fountoulakis KN, Vieta E, Bouras C, *et al*: A systematic review of existing data on long-term lithium therapy: neuroprotective or neurotoxic? *Int J Neuropsychopharmacol* 2008, 11(2):269-287.
55. Kessing LV, Sondergard L, Forman JL, Andersen PK: Lithium treatment and risk of dementia. *Arch Gen Psychiatry* 2008, 65(11):1331-1335.
56. Smith EG, Sondergard L, Lopez AG, Andersen PK, Kessing LV: Association between consistent purchase of anticonvulsants or lithium and suicide risk: a longitudinal cohort study from Denmark, 1995-2001. *J Affect Disord* 2009, 117(3):162-167.

57. Kessing LV, Forman JL, Andersen PK: **Does lithium protect against dementia?** *Bipolar Disord* 2010, **12(1)**:87-94.
58. Warner-Schmidt JL, Duman RS: **Hippocampal neurogenesis: opposing effects of stress and antidepressant treatment.** *Hippocampus* 2006, **16(3)**:239-249.
59. Kessing LV, Sondergard L, Forman JL, Andersen PK: **Antidepressants and dementia.** *J Affect Disord* 2009.
60. Salvadore G, Drevets WC, Henter ID, Zarate CA, Manji HK: **Early Intervention in Bipolar Disorder, Part I: Clinical and Imaging Findings.** *Early Interv Psychiatry* 2008, **2(3)**:122-135.
61. Hirschfeld RM, Lewis L, Vornik LA: **Perceptions and impact of bipolar disorder: how far have we really come? Results of the national depressive and manic-depressive association 2000 survey of individuals with bipolar disorder.** *J Clin Psychiatry* 2003, **64(2)**:161-174.
62. Zwarenstein M, Treweek S, Gagnier JJ, *et al*: **Improving the reporting of pragmatic trials: an extension of the CONSORT statement.** *BMJ* 2008, **337**: a2390.
63. Kessing LV, Hansen HV, Hougaard E, Hvenegaard A, Albæk J: **Preventive outpatient treatment in affective disorders. Results from a Health Technology Assessment (HTA).** Copenhagen: National Board of Health, Danish Centre for Evaluation and Health Technology Assessment Danish Health Technology Assessment - Projects funded by Dacehta; 2006, **6(9)** [http://www.sst.dk/publ/publ2006/CEMTV/Affektive_lidelser/affektive_lidelser_UK.pdf], 2006. Ref Type: Report.
64. Dam H, Bendsen BB, Jakobsen K, *et al*: **Variations between psychiatric hospitals in clinical features and treatment of depression - results from a depression database for inpatients in Copenhagen.** *Ugeskr Laeger* 2010, **172**:3183-3187.
65. Licht RW, Vestergaard P, Kessing LV, Larsen JK, Thomsen PH: **Psychopharmacological treatment with lithium and antiepileptic drugs: suggested guidelines from the Danish Psychiatric Association and the Child and Adolescent Psychiatric Association in Denmark.** *Acta Psychiatr Scand Suppl* 2003, , **419**: 1-22.
66. National Board of Health DS: **Reference program for adults with unipolar depression (in Danish: Referenceprogram for unipolar depression hos voksne).** 2007, Ref Type: Report.
67. Grunze H, Kasper S, Goodwin G, Bowden C, Moller HJ: **The World Federation of Societies of Biological Psychiatry (WFSBP) guidelines for the biological treatment of bipolar disorders, part III: maintenance treatment.** *World J Biol Psychiatry* 2004, **5(3)**:120-135.
68. Grunze H, Kasper S, Goodwin G, *et al*: **World Federation of Societies of Biological Psychiatry (WFSBP) guidelines for biological treatment of bipolar disorders. Part I: Treatment of bipolar depression.** *World J Biol Psychiatry* 2002, **3(3)**:115-124.
69. Grunze H, Vieta E, Goodwin GM, *et al*: **The World Federation of Societies of Biological Psychiatry (WFSBP) Guidelines for the Biological Treatment of Bipolar Disorders: Update 2010 on the treatment of acute bipolar depression.** *World J Biol Psychiatry* 2010, **11(2)**:81-109.
70. Bauer M, Whybrow PC, Angst J, Versiani M, Möller H-J, WFSBP Task Force on Treatment Guidelines for Unipolar Depressive Disorders: **World Federation of Societies of Biological Psychiatry (WFSBP) Guidelines for biological treatment of unipolar depressive disorders, Part 2: Maintenance treatment of major depressive disorder and treatment of chronic depressive disorders and subtreshold depressions.** *World J Biol Psychiatry* 2002, **3**:69-86.
71. Bauer M, Whybrow P, Angst J, Versiani M, Moeller HJ, WFSBP Task Force on Treatment of Unipolar Depressive Disorder: **World Federation of Societies of Biological Psychiatry (WFSBP) Guidelines for Biological Treatment of Unipolar Depressive Disorders, Part 1: Acute and continuation treatment of major depressive disorder.** *World J Biol Psychiatry* 2002, **3**:5-43.
72. Munk-Jorgensen P, Mortensen PB: **The Danish Psychiatric Central Register.** *Dan Med Bull* 1997, **44(1)**:82-84.
73. World Health Organisation: **Klassifikation af sygdomme. Systematisk del. International Statistical Classification of Diseases and Health Related Problems. Tenth revision (Danish Edition).** Copenhagen: Munksgaard, Copenhagen; 1993.
74. Bech P, Olsen LR: **[Discovering depression].** *Ugeskr Laeger* 2001, **163(14)**:1980-1982.
75. Bech P, Rasmussen NA, Olsen LR, Noerholm V, Abildgaard W: **The sensitivity and specificity of the Major Depression Inventory, using the Present State Examination as the index of diagnostic validity.** *J Affect Disord* 2001, **66(2-3)**:159-164.
76. Olsen LR, Jensen DV, Noerholm V, Martiny K, Bech P: **The internal and external validity of the Major Depression Inventory in measuring severity of depressive states.** *Psychol Med* 2003, **33(2)**:351-356.
77. Hirschfeld RM, Williams JB, Spitzer RL, *et al*: **Development and validation of a screening instrument for bipolar spectrum disorder: the Mood Disorder Questionnaire.** *Am J Psychiatry* 2000, **157(11)**:1873-1875.
78. Hirschfeld RM, Holzer C, Calabrese JR, *et al*: **Validity of the mood disorder questionnaire: a general population study.** *Am J Psychiatry* 2003, **160(1)**:178-180.
79. Demyttenaere K, Enzlin P, Dewe W, *et al*: **Compliance with antidepressants in a primary care setting, 2: the influence of gender and type of impairment.** *J Clin Psychiatry* 2001, **62(Suppl 22)**:34-7, 34-37.
80. Kessing LV, Hansen HV, Demyttenaere K, Bech P: **Depressive and bipolar disorders: patients' attitudes and beliefs towards depression and antidepressants.** *Psychol Med* 2005, **35(8)**:1205-1213.
81. Bech P, Olsen LR, Kjoller M, Rasmussen NK: **Measuring well-being rather than the absence of distress symptoms: a comparison of the SF-36 Mental Health subscale and the WHO-Five Well-Being Scale.** *Int J Methods Psychiatr Res* 2003, **12(2)**:85-91.
82. Bech P: **Health-related quality of life measurements in the assessment of pain clinic results.** *Acta Anaesthesiol Scand* 1999, **43(9)**:893-896.
83. Kessing LV, Hansen HV, Ruggeri M, Bech P: **Satisfaction with treatment among patients with depressive and bipolar disorders.** *Soc Psychiatry Psychiatr Epidemiol* 2006, **41(2)**:148-155.
84. Juel K, Helweg-Larsen K: **The Danish registers of causes of death.** *Dan Med Bull* 1999, **46**:354-357.
85. Department of Psychiatric Demography AD: **Annual report 2001.** 2002, Ref Type: Report.
86. Colom F, Vieta E, Martinez-Aran A, *et al*: **A randomized trial on the efficacy of group psychoeducation in the prophylaxis of recurrences in bipolar patients whose disease is in remission.** *Arch Gen Psychiatry* 2003, **60(4)**:402-407.
87. Danish National Board of Health L: **Medicinal Product Statistics.** 2002 [http://www.laegemiddelstyrelsen.dk], Ref Type: Electronic Citation.
88. Kessing L: **Validity of diagnoses and other clinical register data in patients with affective disorder.** *Eur Psychiatry* 1998, **13(8)**:392-398.
89. Bock C, Bukh JD, Vinberg M, Gether U, Kessing LV: **Validity of the diagnosis of a single depressive episode in a case register.** *Clin Pract Epidemiol Ment Health* 2009, **5(4)**:4.
90. Burns T: **End of the road for treatment-as-usual studies?** *Br J Psychiatry* 2009, **195(1)**:5-6.

doi:10.1186/1745-6215-12-32
Cite this article as: Kessing *et al.*: The effects of centralised and specialised combined pharmacological and psychological intervention compared with decentralised and non-specialised treatment in the early course of severe unipolar and bipolar affective disorders - design of two randomised clinical trials. *Trials* 2011 **12**:32.

Submit your next manuscript to BioMed Central and take full advantage of:

- **Convenient online submission**
- **Thorough peer review**
- **No space constraints or color figure charges**
- **Immediate publication on acceptance**
- **Inclusion in PubMed, CAS, Scopus and Google Scholar**
- **Research which is freely available for redistribution**

Submit your manuscript at
www.biomedcentral.com/submit

BioMed Central
